# The comparison of emergency medical service responses to and outcomes of out-of-hospital cardiac arrest before and during the COVID-19 pandemic in Thailand: a cross-sectional study

**DOI:** 10.1186/s12245-023-00489-x

**Published:** 2023-02-20

**Authors:** Thongpitak Huabbangyang, Rossakorn Klaiangthong, Agasak Silakoon, Suttida Sretimongkol, Sutasinee Sangpakdee, Manit Khiaolueang, Pattama Seancha, Tontrakan Nuansamlee, Anucha Kamsom, Ratree Chaisorn

**Affiliations:** 1 Department of Disaster and Emergency Medical Operation, Faculty of Science and Health Technology, Navamindradhiraj University, Bangkok, Thailand; 2 Faculty of Medicine, Vajira Hospital, Navamindradhiraj University, Bangkok, Thailand; 3 Division of Biostatistic, Faculty of Medicine Vajira Hospital, Navamindradhiraj University, Bangkok, Thailand; 4 Division of Emergency Medical Service and Disaster, Faculty of Medicine Vajira Hospital, Navamindradhiraj University, Bangkok, Thailand

**Keywords:** COVID-19, Emergency medical services, Out-of-hospital cardiac arrest, Response time, ROSC

## Abstract

**Background:**

During the coronavirus disease 2019 (COVID-19) pandemic, the format of patients with out-of-hospital cardiac arrest (OHCA) management was modified. Therefore, this study compared the response time and survival at the scene of patients with OHCA managed by emergency medical services (EMS) before and during the COVID-19 pandemic in Thailand.

**Methods:**

This retrospective, observational study used EMS patient care reports to collect data on adult patients with OHCA coded with cardiac arrest. Before and during the COVID-19 pandemic was defined as the periods of January 1, 2018–December 31, 2019, and January 1, 2020–December 31, 2021, respectively.

**Results:**

A total of 513 and 482 patients were treated for OHCA before and during the COVID-19 pandemic, respectively, showing a decrease of 6% (% change difference =− 6.0, 95% confidence interval [CI] *−* 4.1, − 8.5). However, the average number of patients treated per week did not differ (4.83 ± 2.49 vs. 4.65 ± 2.06; *p* value = 0.700). While the mean response times did not significantly differ (11.87 ± 6.31 vs. 12.21 ± 6.50 min; *p* value = 0.400), the mean on-scene and hospital arrival times were significantly higher during the COVID-19 pandemic compared with before by 6.32 min (95% CI 4.36–8.27; *p* value < 0.001), and 6.88 min (95% CI 4.55–9.22; *p* value < 0.001), respectively. Multivariable analysis revealed that patients with OHCA had a 2.27 times higher rate of return of spontaneous circulation (ROSC) (adjusted odds ratio = 2.27, 95% CI 1.50–3.42, *p* value < 0.001), and a 0.84 times lower mortality rate (adjusted odds ratio = 0.84, 95% CI: 0.58–1.22, *p* value = 0.362) during the COVID-19 pandemic period compared with that before the pandemic.

**Conclusions:**

In the present study, there was no significant difference between the response time of patients with OHCA managed by EMS before and during COVID-19 pandemic period; however, markedly longer on-scene and hospital arrival times and higher ROSC rates were observed during the COVID-19 pandemic than those in the period before the pandemic.

## Introduction

Out-of-hospital cardiac arrest (OHCA) results from sudden circulatory collapse and is life-threatening unless prompt cardiopulmonary resuscitation (CPR) is performed with an automated external defibrillator. OHCA is the leading cause of death worldwide, with a global incidence of 50–60 per 100,000 people per year [1]. In the USA, more than 350,000 people die annually due to cardiac arrest [2]. In Asia Pacific, patients with OHCA have a very high mortality rate of 94.6% [3]. In Thailand, the incidence of OHCA averages 6,450 cases per year. More than 65.7% of the patients with OHCA who are successfully resuscitated at the scene by emergency medical service (EMS) survived at the scene [4]; however, their survival rate to admission and discharge is only 27.7% and 4.2%, respectively. For the most common cause of OHCA, more than half was presumed cardiac etiology [5].

In late December 2019, the world faced the outbreak of the coronavirus disease 2019 (COVID-19) infection, which was declared a global pandemic by the World Health Organization. This pandemic has impacted the way of life, quality of life, economy, society, and the global health system [6, 7]. In developed countries, adaptations were attempted to reduce these impacts, such as the declaration of a state of emergency, the stay-at-home order, nationwide lockdown policies, social restrictions, and social distancing [8]. Thailand was one of the first countries to report infection outside China [9] and the impacts and cumulative number of infected people in the country has not yet improved. The Thai government implemented measures to improve the situation, such as curfew declaration for the hours of 10 pm–4 am and the closing of public places, department stores, schools and universities [10].

COVID-19 significantly affected the health system, particularly EMS; for example, the number of patients serviced by EMS and emergency departments in California decreased [11]. These effects led to adjustments in important EMS operation formats, such as additional personal protective equipment (PPE) use, detailing patients’ exposure history, and significant increases in EMS response time [12–14], which significantly improved survival and neurological outcomes [15]. A systematic review and meta-analysis reported that during COVID-19 pandemic, the number of patients with OHCA increased 120%; the response time significantly increased; and increased mortality rate at the scene increased (odds ratio [OR] = 0.67, 95% confidence interval [CI] 0.49–0.91) [16]. Similarly, another study found that while the incidence of OHCA increased, patients’ outcome worsened during COVID-19 pandemic period, compared to that before the pandemic [17]. Substantially decreased bystander CPR was performed for patients with OHCA during the COVID-19 pandemic period compared to that before the pandemic period in Japan [18]. However, few studies have reported on the response time and survival at the scene of patients with OHCA before and during COVID-19 pandemic period in Thailand. To fill this knowledge gap, the present study was conducted to compare the response time and survival at the scene of patients with OHCA before and during the COVID-19 pandemic period.

## Methods

### Study design and setting

This retrospective observational study utilized data collected by the Surgico Medical Ambulance and Rescue Team (S.M.A.R.T), Faculty of Medicine Vajira Hospital, Navamindradhiraj University, Bangkok, Thailand, dispatched by Erawan Center, Bangkok, which has six network hospitals, responsible for the care of 500,000 people within 50 square kilometers [4].The first patient in the study area was confirmed on January 13th, 2020, by the Ministry of Public Health. During the study period, 437,303 patients were confirmed as having COVID-19 in the study area, making it the most densely populated COVID-19 area [19]. During the COVID-19 pandemic, operation formats of the S.M.A.R.T included screening for patients under investigation (PUIs) by paramedics or emergency nurse practitioners (ENPs) via emergency medical hotline, 1554, or order from Bangkok dispatch center, as well as a patient symptom report summary and risk of COVID-19 infection from emergency medical dispatcher. Staff delivering patients with EMS wore PPE and avoided aerosol generating procedures, such as advance airway management and mechanical CPR in patients with OHCA. Each emergency operation team included at least three staff members, composed of emergency physicians, paramedics and ENPs as an operation unit leader, as well as emergency medical technicians (EMTs).

### Study participants

Data of patients with OHCA aged 18 years and older who were dispatched to S.M.A.R.T and who were coded with cardiac arrest by the Thailand Emergency Medical Triage Protocol and Criteria Based Dispatch (CBD) were retrospectively collected from EMS patient care reports for study analysis. Patient with incomplete data or missing data, cardiac arrest outside the scene, OHCA during transfer, termination of resuscitation at the scene, or who were evaluated as dead, deemed unsuitable for resuscitation by the team leader, or denied resuscitation, were excluded from the study. The study period comprised four years; before and during the COVID-19 pandemic was defined as the periods of January 1, 2018–December 31, 2019, and January 1, 2020–December 31, 2021, respectively.

### Data sources and collection

Patient data was retrospectively collected from patients with EMS care report. The form consisted of EMS operation unit data, patient data, and all EMS team’s treatments recorded by the dispatcher and operating EMS staff. The following data was extracted and saved in a Microsoft excel program: (1) OHCA patient characteristics, including age (years), gender, underlying disease, etiology, location, witnessed arrest, bystander CPR performed, bystander AED applied; (2) prehospital management by EMS, including initial arrest rhythm, defibrillation, advanced airway management, prehospital fluid management, medication use during CPR process, ROSC at scene, determination of death at scene; and (3) EMS processing time, including response time (minutes), on-scene time (minutes), transportation time (minutes), and hospital arrival time (minutes).

For the operational definitions, ROSC at the scene was specified as the ability of the heart to pump blood throughout the body, a palpable pulse, and a measurable blood pressure at scene; response time was defined as the duration from emergency call to ambulance arrival at the scene; on-scene time was the duration from ambulance arrival at the scene to ambulance departure from the scene; transportation time was the duration from ambulance departure from the scene to ambulance arrival at the designated hospital; and hospital arrival time was defined as the duration from emergency call to ambulance arrival at the designated hospital.

### Outcome measures

The primary objective was the response time of patients with OHCA managed by EMS before and during COVID-19 pandemic period. The secondary objective was patients’ survival at the scene before and during COVID-19 pandemic period.

### Sample size

Sample size estimation testing two independent means was used for the primary objective [20]. Statistical data used in sample size calculation was described in a previous study [14]. The mean and standard deviation response times before and during the COVID-19 pandemic were 389.7 ± 201.8 and 445.8 ± 210.2 s, respectively. The ratio of the sample size of comparative to studied groups was defined as 1. A level of statistical significance of 0.05 and power of 80% were determined. The calculated sample size was at least 212 per group. Sample size estimation testing two independent proportions was used for the secondary objective [20]. Statistical data used in the sample size calculation also referred to the previous study [14]. Prehospital ROSC at the scene of patients with OHCA before and during COVID-19 pandemic period were 6.49% (p_2_ = 0.0649) and 2.57% (p_1_ = 0.0257), respectively. The ratio of the sample size of comparative to studied groups was defined as 1, according to the population proportion. The calculated sample size was at least 442 per group.

In the present study, sample size was determined as the whole number of patients with OHCA at the scene, serviced by EMS of the S.M.A.R.T who matched the eligibility criteria during the study period. The screened sample size was 995, which was sufficient for analyses.

### Statistical analysis

We performed descriptive analysis to examine the distribution of variables. Continuous variables are presented as the mean ± standard deviation (SD) or median and interquartile range (IQR), and categorical variables are presented as frequencies and proportions. In the comparison of the two groups, we compared the group differences using independent *t* tests or Mann–Whitney *U* tests for numeric variables and chi-square tests or Fisher’s exact tests for categorical variables.

Analyses of the results of COVID-19 pandemic on response time and ROSC at the scene of patients with OHCA with multivariable analysis using multiple logistic regression analysis were reported with OR and 95% CIs. Statistical analyses were performed using IBM SPSS Statistics for Windows, Version 28.0. Armonk, NY, USA: IBM Corp. *P* value at 0.05 were considered significant.

## Results

### General data

In total, data from 482 patients were extracted for the comparison study of EMS response and outcome of patients with OHCA before and during COVID-19 pandemic period in Thailand, the sample was patients with OHCA at scene, assisted by EMS of the S.M.A.R.T, Faculty of Medicine Vajira Hospital, Navamindradhiraj University, categorized as before COVID-19 pandemic period, during 1st January 2018–31st December 2019 and during COVID-19 pandemic period, during 1st January 2020–31st December 2021, with the number of 482. The number of excluded OHCA patients from the present study, before and during the COVID-19 pandemic period, was 54, divided into 23 patients before COVID-19 period, including 16 patients with incomplete data or missing data, 1 patient with cardiac arrest outside the scene, 1 patient with OHCA during transfer, as well as 5 patients with termination of resuscitation at the scene, and 31 patients during COVID-19 period, including 14 patients with incomplete data or missing data, 2 patients with OHCA during transfer, 14 patients with termination of resuscitation at the scene and 1 OHCA patient, not proper for resuscitation evaluated by a team leader or denying resuscitation.

### General data and clinical characteristics of the sample

Before and during COVID-19 pandemic period, mean age of the sample were 64.18 ± 19.94 and 65.18 ± 18.16 years, respectively (*p* value = 0.410) and most were male, 62.4% and 63.1%, consecutively (*p* value = 0.821). Underlying disease was found in 25.7% and 44.6 % of the patients before and during COVID-19 pandemic period, respectively (*p* value < 0.001). The most common etiology was non-traumatic, 95.7% and 91.9%, consecutively (*p* value = 0.012). The most common location was home, 85.6% and 69.7%, respectively (*p* value < 0.001). Witnessed arrests were 44.1% and 88.4%, consecutively (*p* value < 0.001). Bystander CPR was performed 34.3% and 67.8%, respectively (*p* value < 0.001). And bystander AED was applied 5.8% and 8.1%, consecutively (*p* value = 0.164). The most common initial arrest rhythm was asystole, 81.3% and 77.6% before and during COVID-19 pandemic period, respectively (*p* value = 0.126). Defibrillation was performed 10.1% and 17%, consecutively (*p* value = 0.001). Endotracheal intubation was done 46% and 51.9%, respectively (*p* value = 0.003). And the most common fluid management was normal saline solution (NSS), 65.3% and 61.4%, consecutively (*p* value = 0.066). Medication was used 64.1% and 64.5% during CPR process, respectively (*p* value = 0.898), including epinephrine (63.7% and 64.5%, consecutively; *p* value = 0.798), amiodarone (5.8% and 13.5%, respectively; *p* value < 0.001), sodium bicarbonate (15% and 31.5%, consecutively; *p* value < 0.001), glucose (5.3% and 5.4%, respectively; *p* value = 0.927), calcium gluconate (7.8% and 14.3%, consecutively; *p* value = 0.001) and atropine (0.8% and 2.5%, respectively; *p* value = 0.032) (Table 1).

**Table 1:** Patients’ demographic and clinical characteristics

Characteristics	During COVID-19 pandemic		Before COVID-19 pandemic		*p* value
Total	482		513		
% Change Difference (95% CI)	− 6.0		(− 4.1, − 8.5)		
Mean number of patients per week	4.83 ± 2.49		4.65 ± 2.06		0.700
Characteristics of patients with OHCA					
Age (years)	65.18 ± 18.16		64.18 ± 19.94		0.410
Gender					
Male	304	(63.1)	320	(62.4)	0.821
Female	178	(36.9)	193	(37.6)	
Underlying disease					
No	65	(13.5)	5	(1.0)	< 0.001
Yes	215	(44.6)	132	(25.7)	
Unknown	202	(41.9)	376	(73.3)	
Etiology					
Non-traumatic	443	(91.9)	491	(95.7)	0.012
Traumatic	39	(8.1)	22	(4.3)	
Location					
Homes	336	(69.7)	439	(85.6)	< 0.001
Public areas	126	(26.1)	61	(11.9)	
Others	20	(4.1)	13	(2.5)	
Witnessed arrest	426	(88.4)	226	(44.1)	< 0.001
Bystander CPR performed	327	(67.8)	176	(34.3)	< 0.001
Bystander AED applied	39	(8.1)	30	(5.8)	0.164
Prehospital management by EMS					
Initial arrest rhythm					
Asystole	374	(77.6)	417	(81.3)	0.126
VF	58	(12.0)	40	(7.8)	
PEA	44	(9.1)	46	(9.0)	
pVT	6	(1.2)	10	(1.9)	
Defibrillation	82	(17.0)	52	(10.1)	0.001
Advanced airway management					
Endotracheal intubation	250	(51.9)	236	(46.0)	0.003
Supraglottic airway	24	(5.0)	54	(10.5)	
Others	208	(43.2)	223	(43.5)	
Prehospital fluid management					
NSS	296	(61.4)	335	(65.3)	0.066
RLS	12	(2.5)	4	(0.8)	
Others	174	(36.1)	174	(33.9)	
Medication use during CPR process	311	(64.5)	329	(64.1)	0.898
Epinephrine	311	(64.5)	327	(63.7)	0.798
Amiodarone	65	(13.5)	30	(5.8)	< 0.001
Sodium bicarbonate	152	(31.5)	77	(15.0)	< 0.001
Glucose	26	(5.4)	27	(5.3)	0.927
Calcium gluconate	69	(14.3)	40	(7.8)	0.001
Atropine	12	(2.5)	4	(0.8)	0.032

*Abbreviations*: *CI* confidence interval, *CPR* cardiopulmonary resuscitation, *AED* automated external defibrillator, *VF* ventricular fibrillation, *PEA* pulseless electrical activity, *pVT* pulseless ventricular tachycardia, *NSS* normal saline solution, *RLS* ringer’s lactate solution. Data are presented as number (%), mean ± standard deviation. *P* value corresponds to independent samples *t* test or chi-square test

**Fig. 1:**
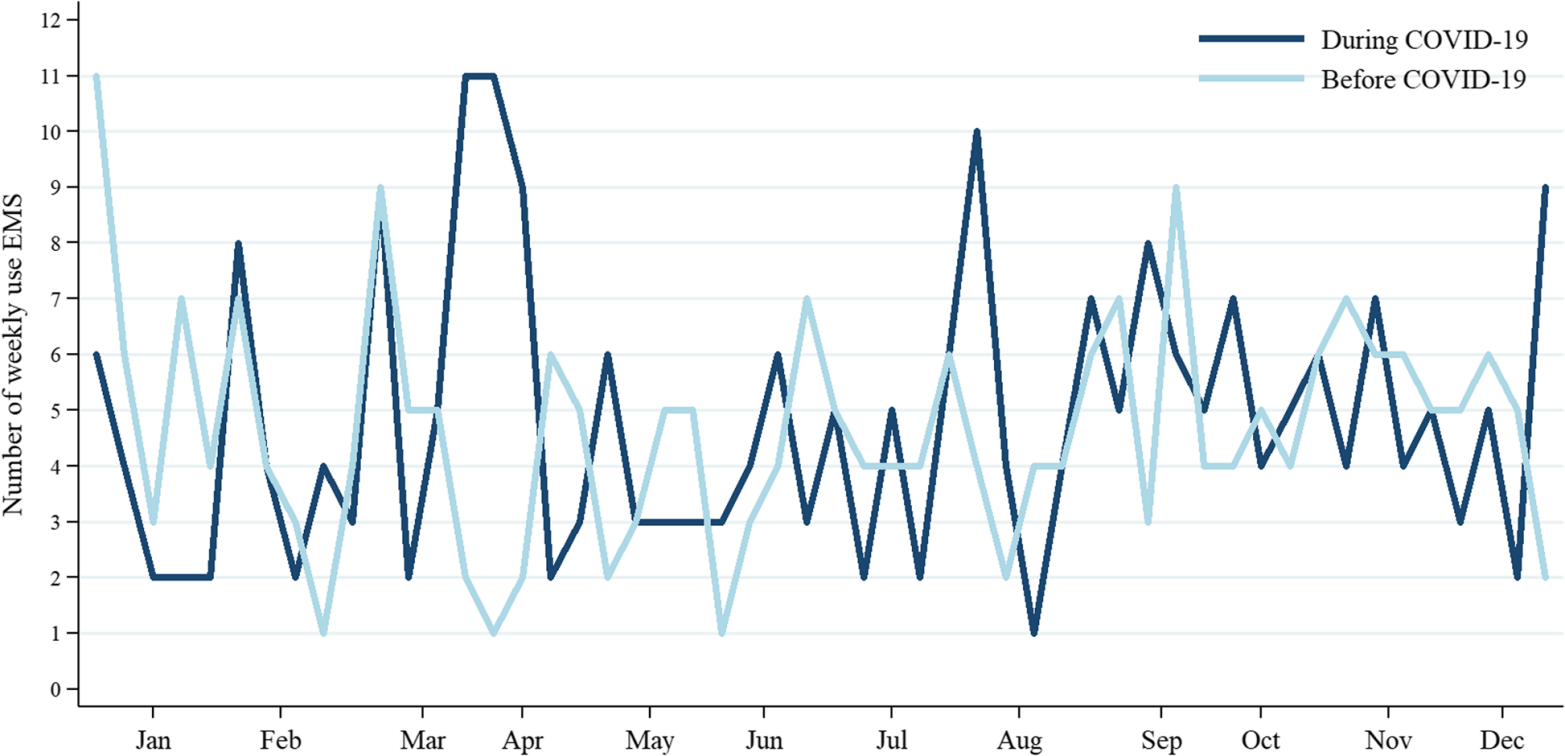
Comparison of the number of patients with OHCA per week serviced by EMS before and during the COVID-19 pandemic

In total, 513 and 482 patients were treated before and during the COVID-19 pandemic, resulting in a decrease of 6% (% Change Difference = − 6.0, 95%CI − 4.1, − 8.5). However, the average number of the patients per week did not differ between before and during the pandemic (4.65 ± 2.06 vs. 4.83 ± 2.49, respectively; *p* value = 0.700) (Fig. 1 and Table 1).

### Comparison of response time in patients with OHCA before and during COVID-19 pandemic period

**Fig. 2:**
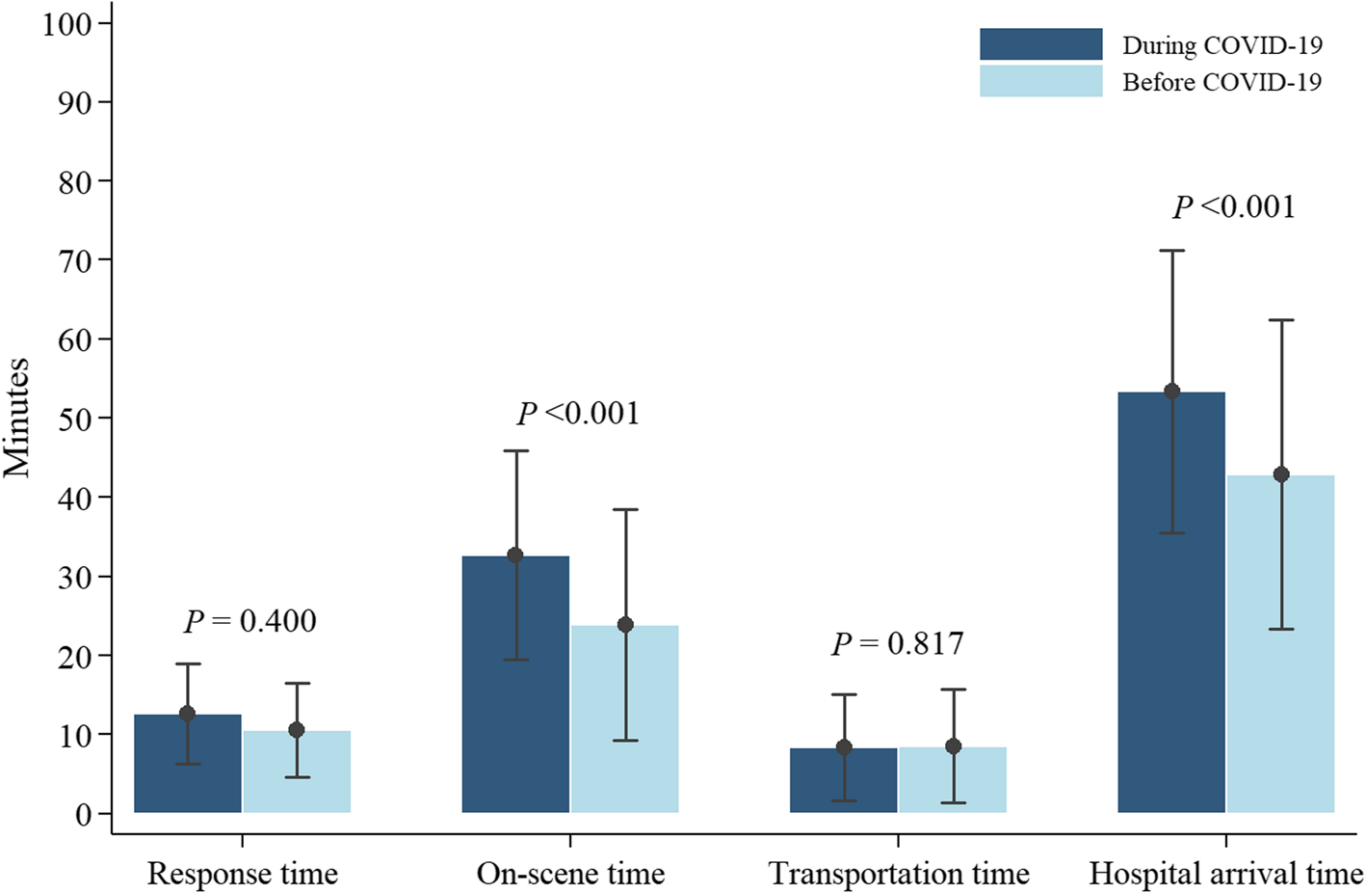
Comparison of the mean error bar EMS processing time, including response, on-scene, transportation, and hospital arrival times of patients with OHCA before and during COVID-19 pandemic

Table 2 and Fig. 2 depict the mean EMS processing time, including the response, on-scene, transportation, and hospital arrival times for patients with OHCA before and during the COVID-19 pandemic. There was no significant difference in the mean response time of patients with OHCA before and during the COVID-19 pandemic (11.87 ± 6.31 vs.12.21 ± 6.50 min, respectively; *p* value = 0.400). However, the mean on-scene time during the COVID-19 pandemic was significantly higher than that before it at 29.83 ± 16.63 and 23.51 ± 14.77 min, respectively (increase of 6.32 min 95% CI 4.36–8.27; *p* value < 0.001).

**Table 2:** Response time for patients with out-of-hospital cardiac arrest (OHCA) before and during the COVID-19 pandemic

EMS processing time	During COVID-19 pandemic (*n* = 482)	Before COVID-19 pandemic (*n* = 513)	Mean difference	95%CI	*p* value
	Mean ± SD	Mean ± SD			
Response time (minutes)	12.21 ± 6.50	11.87 ± 6.31	0.34	(− 0.46 to 1.14)	0.400
On-scene time (minutes)	29.83 ± 16.63	23.51 ± 14.77	6.32	(4.36 to 8.27)	< 0.001
Transportation time (minutes), ^a^(*n* = 340)	8.38 ± 6.73	8.56 ± 7.15	− 0.17	(− 1.65 to 1.31)	0.817
Hospital arrival time (minutes)	44.89 ± 19.7	38.01 ± 17.76	6.88	(4.55 to 9.22)	< 0.001

*Abbreviations*: *CI* confidence interval, *SD* standard deviation, *COVID-19* coronavirus disease 2019. *P* value corresponds to independent samples *t* test

Mean transportation time before and during the COVID-19 pandemic was 8.56 ± 7.15 and 8.38 ± 6.73 min, respectively, which was not statistically different (*p* value = 0.817). However, the mean hospital arrival time during the COVID-19 pandemic was 6.88 min longer than that before at 44.89 ± 19.7 and 38.01 ± 17.76 min, respectively (95% CI 4.55–9.22; *p* value < 0.001)

### Comparison of ROSC at scene in patients with OHCA before and during COVID-19 pandemic period

The ROSC rate before and during the COVID-19 pandemic was 14.0% and 27.8%, respectively (*p* value < 0.001), while mortality rate at the scene was 73.9% and 71.0% (*p* value = 0.302) (Table 3).

**Table 3:** Return of spontaneous circulation (ROSC) at scene for out-of-hospital cardiac arrest (OHCA) before and during the COVID-19 pandemic period

Outcome	During COVID-19 pandemic (*n* = 482)		Before COVID-19 pandemic (*n* = 513)		*p* value
	*n*	(%)	*n*	(%)	
ROSC at scene					
No	348	(72.2)	441	(86.0)	< 0.001
Yes	134	(27.8)	72	(14.0)	
Determination of death at scene					
No	140	(29.0)	134	(26.1)	0.302
Yes	342	(71.0)	379	(73.9)	

*Abbreviations*: *ROSC* return of spontaneous circulation, *COVID-19* coronavirus disease 2019

*P* value corresponds to chi-square test

For univariable analysis of ROSC using simple logistic regression analysis, patients during COVID-19 pandemic period had a 2.36 times higher ROSC rate (OR = 2.36, 95% CI 1.71–3.25, *p* value < 0.001) and an 0.86 times decrease in mortality rate at the scene (OR = 0.86, 95%CI 0.65–1.14, *p* value = 0.302) during the COVID-19 pandemic compared with that before the pandemic (Table 4).

**Table 4:** Multivariable analysis of the return of spontaneous circulation (ROSC) at the scene before and during the COVID-19 pandemic

	Univariable analysis			Multivariable analysis		
	OR^a^	95%CI	*p* value	OR_adj_^b^	95%CI	*p* value
ROSC at scene						
During COVID-19 pandemic	2.36	(1.71–3.25)	< 0.001	2.27	(1.50–3.42)	< 0.001
Before COVID-19 pandemic	1.00	Reference		1.00	Reference	
Determination of death at scene						
During COVID-19 pandemic	0.86	(0.65–1.14)	0.302	0.84	(0.58–1.22)	0.362
Before COVID-19 pandemic	1.00	Reference		1.00	Reference	

*Abbreviations*: *OR* odds ratio, *OR*_*adj*_ adjusted odds ratio, *CI* confident interval, *ROSC* return of spontaneous circulation, *COVID-19* coronavirus disease 2019

^a^Crude odds ratio estimated by binary logistic regression

^b^Adjusted odds ratio estimated by multiple logistic regression adjusted for age, gender, underlying disease, etiology, location, witnessed arrest, bystander CPR performed, bystander AED applied, initial arrest rhythm, defibrillation, advanced airway management, prehospital fluid management, and medication use during CPR process

For multivariable analysis using multiple logistic regression analysis, patients with OHCA had a 2.27 times higher rate of ROSC (adjusted OR = 2.27, 95% CI 1.50–3.42; *p* value < 0.001) and a 0.84 times lower rate of mortality (adjusted OR = 0.84, 95% CI 0.58–1.22, *p* value = 0.362) during the COVID-19 pandemic than before it, after controlling for confounders, including age, gender, underlying disease, etiology, location, witnessed arrest, bystander CPR performed, bystander AED applied, initial arrest rhythm, defibrillation, advanced airway management, prehospital fluid management, and medication use during the CPR process (Table 4).

## Discussion

Firstly, the results of this study showing the impact of COVID-19 in patients with OHCA requiring EMS in Thailand show that before the COVID-19 pandemic, there were a higher number of patients with OHCA than during COVID-19 pandemic period. This contrasts the results of studies in developed countries, such as Singapore and the USA, where the number of patients with OHCA serviced by EMS increased during the COVID-19 pandemic [13, 21], and a systematic review and meta-analysis in Australia showed that the incidence of OHCA 120% compared to that before the pandemic [16]. The decreased number of patients with OHCA serviced by EMS during the COVID-19 pandemic observed in this study might be due to OHCA witnesses avoiding choosing EMS or being anxious with the situation of COVID-19 infection in hospitals. A previous study confirmed that during the COVID-19 pandemic, there was an increased trend of witnesses choosing not to assist patients with OHCA and a dramatically decreased out-of-hospital bystander CPR rate, compared to that observed prior to the pandemic [13]. Another study reported shocking findings that witnesses were unwilling to make emergency calls and assist patients with OHCA during the COVID-19 pandemic period [18].

Secondly, we found no statistical difference in the mean response time of patients with OHCA serviced by EMS before and during the COVID-19 pandemic. This result seems to conflict with the results of many previously published studies, including studies in Singapore [13], Taiwan [14], Saudi Arabia [15], England [22], and California [23], which demonstrated a direct effect to increased EMS response time for patients with OHCA during the COVID-19 pandemic. Although all aforementioned studies would examine response time in OHCA patients in just early period of large COVID-19 pandemic or study in short term, only some periods with the highest national cumulative number of COVID-19 patients reported for few months which was different from the present study, examining impacts of COVID-19 on response time in OHCA patients in long term for 2 years in Thailand during COVID-19 pandemic. Therefore, interpretation of results needs to be discreet substantially, mainly considering study period.

Moreover, a systematic review and meta-analysis confirmed that during COVID-19 pandemic period, response time increased [16]. The average response time observed in this study before and during the COVID-19 pandemic probably did not differ because the operation policy, format, and protocol of the EMS system determined that operating staff had to wear PPE while in the ambulance, preparing the team and equipment for immediate response to patients with OHCA, as well as the quality assurance policy of the institute, which used response time as an assessment indicator, possibly leading to no impact on response time, despite the COVID-19 pandemic. In addition, an artificial intelligence call center system was utilized in the Bangkok dispatch center (Erawan Center). When patients or people make emergency calls using the 1669 hotline, the initial selection would divide patients into two groups, one for emergency calls and the other for patients with COVID-19. This would decrease the duration, help separate emergency patients from patients with COVID-19 and decrease the response time. If other processing time was considered, such as on-scene time and hospital arrival time, in the present study, response times were substantially affected, comparable to the previous studies, making the mean during the COVID-19 pandemic statistically higher than that before it [13–15, 22, 23]. This higher response time was partly from the duration at the scene increasing due difficulty in managing and treating patients with OHCA during the COVID-19 pandemic, as well as the local protocol requiring EMS units to resuscitate patients until ROSC before delivery to hospital. During COVID-19 pandemic period, the EMS team modified the OHCA patient response format; apart from PPE use, history taking regarding the risk of PUIs of patients and relatives before entering the scene and application of automated CPR, instead of chest compression by humans, were included. Furthermore, due to having the highest number of infected individuals in Thailand, the emergency department was frequently temporarily closed. Therefore, ambulance delivery duration depended on the capacity of the designated hospitals. This was an important reason for the increase in the hospital arrival time.

Thirdly, during the COVID-19 pandemic, patients with OHCA had a 2.27 times higher ROSC rate and a lower mortality rate than those before the COVID-19. The increased ROSC rate seen in the present study may have from the EMS team being confident when encountering patients with COVID-19 due to PPE use, as well as other important factors affecting the ROSC of patients with OHCA at the scene [4], including witnessed arrest (88.4% during COVID-19 pandemic vs. 44.1% before it), bystander CPR (67.8% during COVID-19 pandemic vs. 34.3% before it), and advanced airway management included endotracheal intubation (51.9% during COVID-19 pandemic vs. 46.0% before it). During the period, EMS teams encountered patients with OHCA with preparation, defense, and adaptation in response to the situation to improve the patients’ survival, so that even though this was the area most impacted by the COVID-19 pandemic situation in Bangkok, Thailand, the quality and service for patients with OHCA was not compromised.

In the present study, there was surprising increase of proportion of bystander CPR during COVID-19 period which was 67.8%, compared to before COVID-19 period which was only 34.3%. The explanation might be partly due to the increased number of witnessed OHCA patients during COVID-19 pandemic period of 88.4%, compared to before the period, the proportion of witnessed OHCA patients decreased 44.1%. Therefore, the increased proportion of witnessed arrest patients affected the increase of bystander CPR. Besides, in the study area which was Bangkok, during COVID-19 pandemic period, when emergency medical dispatcher (EMD) could detect OHCA, he/she would primarily advise bystanders at scene including teaching how to do dispatcher-assisted cardiopulmonary resuscitation (DACPR) to relatives or bystanders before ambulance arrival at the scene. In addition, in the study area, there was CPR training for non-health care providers, such as motorbike riders, the police, soldiers or interested people. The CPR training program included an applied program during COVID-19 pandemic period. This might be the reason why during COVID-19 pandemic period, proportion of bystander CPR in Bangkok, Thailand increased in the present study. Early bystander CPR should be extremely encouraged, because this exceedingly directly affected survival outcome, even with increased on scene-time.

COVID-19 made an important changing phenomenon. AHA has launched standard guidelines for Basic and Advanced Cardiac Life Support with Suspected or Confirmed COVID-19 [24]. The major change from the previous version includes minimization of the number of CPR providers and quick use of mechanical CPR to reduce a risk of aerosol droplet transmission. CPR provision has a high risk of COVID-19 infection. Consistent with European Resuscitation Council (ERC) COVID-19 guidelines supporting mechanical CPR instead of original manual CPR [17], because there were many problems in the original manual CPR during COVID-19 period, such as difficulty in PPE use and exhaustion of CPR provider. During or even after COVID-19 pandemic period, extensive increase of mechanical CPR could be implied.

The study has a few limitations. Firstly, the database of patients with OHCA was limited, comprising only the treatment information at the scene by the EMS team, and lacked information regarding treatment in the emergency department in hospital. Therefore, the survival outcome was limited to only ROSC at the scene. Secondly, only one place was studied. Hence, although the outcome could be generally applied for the same or similar setting which was substantially affected by the COVID-19 pandemic, the results might not be generally applicable in other contexts. Thirdly, 2 periods were compared (a total of 4 years). The first 2 years were defined as before COVID-19 period which was during 1 January 2018–31 December 2019 and subsequent 2 years was defined as during COVID-19 period which was during 1 January 2020–31 December 2021. However, in fact, direct and indirect impacts of COVID-19 on OHCA patients were substantially different between these periods (e.g., the first two months of the pandemic vs. more recent time periods). This comparison regarding calendar year was to reduce bias from seasonal variation which was a major change in accordance with response of EMS in OHCA patients of both periods in the study area (such as additional history taking about PUI risk before departure, PPE use and mechanical CPR application, instead of manual chest compressions). These might need to be considered in the present study. Fourth, in the area where data were collected, there is no standard form of data collection of OHCA patients according to Utstein-style guidelines. Fifth, in the present study, traumatic cardiac arrest patients were analyzed together with non-traumatic cardiac arrest patients, which might significantly affect study outcomes. Even though, in the present study, the number of traumatic cardiac arrest patients were reported as 39 patients (8.1%) and 22 patients (4.3%) during and before the period, respectively, which were minority.

Lastly, the present study was an observational study; consequently, the impacts of COVID-19 on response time and ROSC at the scene of patients with OHCA could probably not be summarized in the overall image. As a result, prospective and population based studies, as well as qualitative study, are needed in the future to determine the causes properly.

## Conclusion

In the present study, there was no significant difference between the response time of patients with OHCA managed by EMS before and during COVID-19 pandemic period; however, markedly longer on-scene and hospital arrival times and higher ROSC rates were observed during the COVID-19 pandemic than those in the period before the pandemic.

## Data Availability

The datasets generated during and/or analyzed during the current study are available from the corresponding author on reasonable request.

## References

[1] Doctor NE, Ahmad NS, Pek PP, Yap S, Ong ME (2017). The Pan-Asian Resuscitation Outcomes Study (PAROS) clinical research network: what, where, why and how. Singap Med J.

[2] Benjamin EJ, Muntner P, Alonso A, Bittencourt MS, Callaway CW, Carson AP (2019). Heart Disease and Stroke Statistics-2019 Update: A Report From the American Heart Association. Circulation..

[3] Liu N, Ong MEH, Ho AFW, Pek PP, Lu TC, Khruekarnchana P (2020). Validation of the ROSC after cardiac arrest (RACA) score in Pan-Asian out-of-hospital cardiac arrest patients. Resuscitation..

[4] Huabbangyang T, Soion T, Promdee A, Nguanjinda K, Chamchan A, Chaisorn R (2021). Factors associated with successful resuscitation during out-of-hospital cardiac arrest performed by Surgico Medical Ambulance and Rescue Team (S.M.A.R.T), Division of Emergency Medical Service and Disaster, Faculty of Medicine Vajira Hospital, Navamindrad. J Med Assoc Thail.

[5] Ong ME, Shin SD, De Souza NN, Tanaka H, Nishiuchi T, Song KJ (2015). Outcomes for out-of-hospital cardiac arrests across 7 countries in Asia: the Pan Asian Resuscitation Outcomes Study (PAROS). Resuscitation..

[6] Chan JF, Kok KH, Zhu Z, Chu H, To KK, Yuan S (2020). Genomic characterization of the2019novel human-pathogenic coronavirus isolated from a patient with atypical pneumonia after visiting Wuhan. Emerg Microbes Infect.

[7] Li Q, Guan X, Wu P, Wang X, Zhou L, Tong Y (2020). Early transmission dynamics in Wuhan, China, of novel coronavirus-infected pneumonia. N Engl J Med.

[8] Wongtanasarasin W, Srisawang T, Yothiya W, Phinyo P (2021). Impact of national lockdown towards emergency department visits and admission rates during the COVID-19 pandemic in Thailand: A hospital-based study. Emerg Med Australas.

[9] Thu TPB, Ngoc PNH, Hai NM (2020). Effect of the social distancing measures on the spread of COVID-19 in 10 highly infected countries. Sci Total Environ.

[10] Thailand, Ministry of Public Health. Department of Disease Control. Corona Virus Disease (COVID-19) [Internet]. 2020 [cited 2021 Sep 1]. Available from: https://ddc.moph.go.th/viralpneumonia/eng/situation.php.

[11] Rollman JE, Kloner RA, Bosson N, Niemann JT, Gausche-Hill M, Williams M (2021). Emergency medical services responses to out-of-hospital cardiac arrest and suspected ST-segment-elevation myocardial infarction during the COVID-19 pandemic in Los Angeles County. J Am Heart Assoc.

[12] Borkowska MJ, Smereka J, Safiejko K, Nadolny K, Maslanka M, Filipiak KJ (2021). Out-of-hospital cardiac arrest treated by emergency medical service teams during COVID-19 pandemic: A retrospective cohort study. Cardiol J.

[13] Lim SL, Shahidah N, Saffari SE, Ng QX, Ho AFW, Leong BS (2021). Impact of COVID-19 on out-of-hospital cardiac arrest in Singapore. Int J Environ Res Public Health.

[14] Yu JH, Liu CY, Chen WK, Yu SH, Huang FW, Yang MT (2021). Impact of the COVID-19 pandemic on emergency medical service response to out-of-hospital cardiac arrests in Taiwan: a retrospective observational study. Emerg Med J.

[15] Alsofayan YM, Althunayyan SM, Mohamed MA, Alhabeeb SH, Altuwaijri MI, Alhajjaj FS (2021). Out of hospital cardiac arrest: Saudi Red Crescent Experience Throughout COVID-19 Era. Open Access Emerg Med.

[16] Lim ZJ, Ponnapa Reddy M, Afroz A, Billah B, Shekar K, Subramaniam A (2020). Incidence and outcome of out-of-hospital cardiac arrests in the COVID-19 era: A systematic review and meta-analysis. Resuscitation..

[17] Nolan JP, Monsieurs KG, Bossaert L, Böttiger BW, Greif R, Lott C (2020). European Resuscitation Council COVID-19 guidelines executive summary. Resuscitation..

[18] Nishiyama C, Kiyohara K, Iwami T, Hayashida S, Kiguchi T, Matsuyama T (2021). Influence of COVID-19 pandemic on bystander interventions, emergency medical service activities, and patient outcomes in out-of-hospital cardiac arrest in Osaka City, Japan. Resusc Plus.

[19] Ministry of Public Health. Department of Disease Control. Data COVID-19 [Internet]. 2021. [cited 2022 Apr 1]. Available from: https://media.thaigov.go.th/uploads/public_img/source/311264.pdf.

[20] Bernard R (2000). Fundamentals of biostatistics.

[21] Kovach CP, Perman SM (2021). Impact of the COVID-19 pandemic on cardiac arrest systems of care. Curr Opin Crit Care.

[22] Fothergill RT, Smith AL, Wrigley F, Perkins GD (2021). Out-of-Hospital Cardiac Arrest in London during the COVID-19 pandemic. Resusc Plus.

[23] Uy-Evanado A, Chugh HS, Sargsyan A, Nakamura K, Mariani R, Hadduck K (2021). Out-of-hospital cardiac arrest response and outcomes during the COVID-19 pandemic. JACC Clin Electrophysiol.

[24] Atkins DL, Sasson C, Hsu A, Aziz K, Becker LB, Berg RA (2022). 2022 Interim Guidance to Health Care Providers for Basic and Advanced Cardiac Life Support in Adults, Children, and Neonates With Suspected or Confirmed COVID-19: From the Emergency Cardiovascular Care Committee and Get With The Guidelines-Resuscitation Adult and Pediatric Task Forces of the American Heart Association in Collaboration With the American Academy of Pediatrics, American Association for Respiratory Care, the Society of Critical Care Anesthesiologists, and American Society of Anesthesiologists. Circ Cardiovasc Qual Outcomes.

